# Clarifying the muddy concept of home healthcare coordination: A comprehensive theoretical framework

**DOI:** 10.1016/j.heliyon.2023.e14243

**Published:** 2023-03-03

**Authors:** Nathalie Möckli, J. Alberto Espinosa, Michael Simon, Carla Meyer-Massetti, Franziska Zúñiga

**Affiliations:** a Nursing Science, Department of Public Health, University of Basel, Bernoullistrasse 28, CH-4056 Basel, Switzerland; b Kogod School of Business, American University, 4400 Massachusetts Avenue, Washington, DC, NW, 20016, USA; c Clinical Pharmacology & Toxicology, Clinic for General Internal Medicine, Inselspital - University Hospital of Bern, Freiburgstrasse, CH-3010 Bern, Switzerland; d Institute of Primary Health Care (BIHAM), University of Bern, Mittelstrasse 43, CH-3012 Bern, Switzerland

**Keywords:** Care coordination, Coordination, Delivery of health care [mesh], Theoretical framework, Home care services [mesh], Theory, Theoretical models [mesh]

## Abstract

Effective healthcare coordination is vital when such care is provided as a collaborative effort by many individuals and their task activities are interdependent. Coordination is necessary to ensure that care not only meets the needs of patients, but also avoids negative consequences for them due to omitted, inefficient, unnecessary, or even incorrect treatments. It also helps conserve resources. This has contributed to a rapid increase in articles on this subject.

Still, while care coordination topics are gaining the attention of researchers, there are a number of issues experienced, including the delineation of limitations, inconsistent definitions, and problems with measurement. Therefore, the aim of this article is to refine the concept of homecare coordination and provide a comprehensive theoretical framework, illustrated with examples from practice.

Focusing on this goal, we have reviewed the extant literature on the subject to develop a theoretical homecare coordination framework. The first intermediary goal was to integrate relevant concepts across multiple theories and frameworks into a unified synthesis. We do so in two parts: (1) analysis of extant coordination frameworks and theories; and (2) the presentation of our newly developed theoretical framework for homecare coordination.

The new framework differentiates clearly between coordination as a process—i.e., what people do to coordinate and coordination as an outcome—i.e., the state of coordination. Applying this distinction to both, measurement and interpretation of results helps avoid misleading conclusions. As a research outcome, our framework builds upon the extant coordination literature, considers the complex relationships among the various coordination-related factors and, while focusing on homecare, is applicable to various healthcare settings in general.

A nuanced differentiation and explanation of the elements involved enable a more consistent operationalization of the coordination concept. Additionally, as they explicitly address the healthcare system's micro, meso, and macro levels, they can be applied across diverse healthcare settings to investigate homecare coordination.

## Highlights

• Care coordination is essential to meet the needs of patients.
• The lack of clear care coordination conceptualization hinders research.
• To overcome this problem, we developed a theoretical care coordination (COORA) framework.
• COORA distinguishes the different elements of the coordination process and the achieved level of coordination.
• Distinguishingcoordination processes, (coordinating) from coordination as outcome of the process can guide future research.

## 1 Introduction

As the number of people with multiple chronic conditions and the options and specializations for medical treatment increase, the many fields of health care are becoming both more complex and increasingly interdependent [[1], [2], [3]]. As a result, there is a burgeoning need to align the different disciplines and professionals who provide patient care [4] and manage task dependencies among the various health care specialties involved, making care coordination, especially in homecare, particularly important.

Effective care coordination helps care providers deliver patient-centered care that meets patients' needs, largely by avoiding scheduling conflicts and simplifying the management of task dependencies. By reducing omitted, inefficient, unnecessary, or even incorrect treatment—any of which can lead to negative patient outcomes—efficient care coordination avoids both unnecessary patient burden and resource waste [4]. Consequently, care coordination is a fast-evolving research field and is identified as a priority in various countries’ health care strategies (e.g., Switzerland, Canada, Norway) [5].

The conceptualization and measurement of care coordination in and across healthcare settings remains a challenge; and the blending of different concepts and models under the umbrella of “coordination” is pervasive in the literature. A recent example is a scoping review by Peterson et al. [6], which presents integrated care models (e.g., Wagner's Chronic Care Model, Singer's Integrated Patient Care Model), quality models (e.g., Quality Framework by Donabedian), and coordination models (e.g., Gittell's approach to relational coordination, Weaver's multilevel care coordination framework) all under the heading of “Healthcare Coordination Theoretical Frameworks.” These frameworks are very useful and a step in the right direction. However, this eclectic collection of models and concepts comingle multiple perspectives that are not necessarily related to coordination. This makes it difficult to delimit and apply the appropriate concepts from these various related and partly overlapping concepts (e.g., integrated care, quality of care, communication, collaboration). While all of these models include components of care coordination, they differ widely in focus and perspective (e.g., they may focus on quality of care or integrated care rather than care coordination per se). For a reader, such “muddiness” could be confusing or even frustrating. However, for a research team operationalizing the concept of care coordination for a study, a clear understanding and definition of the concept—one that distinguishes it from its nearest neighbors—is essential to avoid misinterpretation and research waste.

Research in coordination in healthcare faces a variety of issues. The first is that, depending on the context in which it is used, coordination may be interchangeable with terms such as teamwork, collaboration, or integration. Such lexical instability can complicate efforts to assess and compare studies and their results [7,8]. In addition, coordination is often mixed with similar concepts such as cooperation, the care process, or case management [7,9,10]. As a result, studies may include “coordination” in their titles, but actually assess related concepts or elements such as leadership skills.

The second problem is a lack of agreement on the multiple definitions of coordination, some of which are so vague that they demand interpretation. In addition, where summaries and descriptions of coordination elements (e.g., coordination mechanisms) are overly broad, they make it difficult to operationalize coordination, which is essential to make it measurable in clinical settings [7,9,11]. One result is that, as one recent review reported, researchers hoping to examine care coordination often had difficulty finding enough published evidence to identify its key elements [8].

The third and biggest problem involves measurement. In this case, instruments developed to measure coordination rather measure care continuity or other related concepts [7]. In addition, they make no distinction between coordination as a process and coordination as an outcome [10,12]. When measuring and analyzing coordination such a distinction is essential: confusing the two can lead to incorrect measurements and conclusions. Nevertheless, recent reviews show that studies commonly measure variables like re-hospitalization rates, patient satisfaction, or other care quality indicators as outcomes of the coordination process—without treating the coordination as an outcome itself—i.e., the level or state of coordination reached as a result of the intermediary coordination processes [8,13]. Establishing a direct link between elements of coordination as a process (e.g., regular meetings) and quality of care outcomes poses problems. It cannot be assumed that simply having coordination processes in place is an assurance that higher levels of coordination (as outcome) will be reached. If they happen to be in place, they will not automatically lead to improved levels of coordination success. For example, there may be a mismatch in the type of coordination process employed and the characteristics of the task. In addition, high levels of coordination (as an outcome) do not necessarily lead to the desired quality outcomes [10,12].

Given the problems outlined above, this article aims to refine the concept of coordination aiming to provide a comprehensive theoretical framework to capture homecare coordination. As we develop the framework, we illustrate its various aspects with practical examples, which will provide the basis for the operationalization and measurement of homecare coordination.

## 2 Methods

Our central component is the development of a comprehensive theoretical homecare coordination framework, aiming to integrate relevant concepts across multiple theories [14,15]. As noted in the extant literature, “a theoretical framework is a structure that summarizes concepts and theories, which are developed from previously tested and published knowledge” (p.46) [15]. To produce this framework, we conducted a literature search of coordination models, frameworks, and theories. Due to the fuzzy nature of the concept of coordination and given the various extant systematic reviews, we decided to build upon these reviews. More specifically, we used four comprehensive coordination literature reviews—by Van Houdt et al. [16], Schultz and McDonald [7], Weaver et al. [13], and Peterson et al. [6] as a starting point to better understand the concept of coordination and the current state of coordination-relevant knowledge in healthcare.

As a first step, we screened the studies referenced in these four literature reviews. We then identified additional studies and related article by searching Medline via Pubmed®. Next, we extended the literature search to include coordination in other research fields (e.g., psychology, sociology, software development) by iteratively searching studies referenced in identified articles using Google Scholar. We then searched new aspects described in relation to the various models and theories—i.e., different elements/underlying concepts of coordination (e.g., communication, mental models)—via Pubmed® and Google Scholar to gain a stronger understanding of underlying or related concepts as we elaborated our framework.

In parallel with the literature search, which was conducted from March 2020 until March 2021, we iteratively discussed and evaluated the models, frameworks, and concepts within the research group. In addition, to deepen our understanding and include a range of expertise in the development of our new theoretical framework, we held discussions with two authors of earlier coordination frameworks [9,12].

Since the healthcare field is very broad and diverse, we focused in this paper more specifically on homecare in order to connect theoretical concepts with practical examples. We view homecare in this study as formal care provided to patients at their homes, including not only medical and therapeutic care but also basic care (e.g., personal hygiene or mobilization), domestic support (e.g., help with shopping, washing or cleaning) and social care (e.g., assistance with making appointments or going for a walk). To better understand the context in general and to gain familiarity about the context of the focal task, we held discussions with different homecare nurses, homecare nursing experts, and homecare patients and their relatives about their understanding of coordination and possible interrelationships with other elements and concepts. We purposely selected nursing experts because of their experience in this field and invited them for discussions and focus groups. Homecare patients, their relatives and nurses were also invited to focus groups by two homecare organizations in consultation with the first author.

## 3 Analysis of coordination frameworks and theories

Our literature review revealed that there are conditions that make coordination necessary and determine the extent to which it is needed. Therefore, we divided this section into two parts.

(1) preconditions for the need for coordination and
(2) the coordination literature, with its definitions, frameworks, and theories.

### 3.1 Preconditions for coordination

Before elucidating the different elements of coordination, it is important to consider that not all work can benefit from coordination. We identified three key preconditions to coordination.

(1) **Task activities have dependencies.** The most important consideration is that task activities included in the work must contain dependencies*.* Naturally, because task activities are carried out by individuals, task dependencies will inevitably lead to member dependencies. Without dependencies, there is really nothing to coordinate [17] and coordination could even be a distraction from the focal task or a cost-ineffective undertaking. Van de Ven et al. [18] described four bases of dependency between participants: 1. The task (workflow between participants); 2. Individual roles (participants position in joint action); 3. Social dependence (participants' reliance upon one another to fill mutual needs or achieve common goals); and 4. Knowledge dependence (participants' reliance on one another's various levels of expertise). Regarding task dependencies, three categories are relevant to coordination: pooled, sequential, and reciprocal [18,19].

For *pooled dependencies****,*** each participant contributes their part somewhat independently or with little and no direct interaction, but depends on a pool of shared resources, such as shared budgets, technical resources, health insurance, etc. [20,21].

*Sequential dependencies* occur when one participant depends on another to complete their part (e.g., medication prescription must be provided before medication can be obtained and taken; and a budget must be approved before the funds can be spent), but the other participant does not depend on one [[20], [21], [22], [23]].

*Reciprocal dependencies or interdependencies* arise when a task requires different participants to undertake different parts of it in a cyclic give-and-take. This interdependence implies some flexibility in the sequencing of tasks; and due to the cyclical nature of the work, adjustments are made iteratively between participants [20,21,23]. For example, adjustment of pain management requires iterative activities between the patient (feedback on whether pain medication is effective), nurse (preparation/administration of pain medication and monitoring of effectiveness), physician (adjustments to pain medication prescription). As dependencies increase in complexity, from pooled to sequential to reciprocal, the need for coordination increases [18]. In the homecare setting, we are mostly confronted with sequential dependence and/or reciprocal dependence [18,21].

(2) **There are multiple participants** involved in the work. Even if task activities have dependencies, if there is only a single individual carrying out the task, there is no need for members to coordinate [18]. That is, the lower the proportion of one-person tasks and the higher the degree of task-related cooperation, the higher the dependence level [18]. The dependency grows exponentially to the number of persons [24]. For example, a group with *n* members collaborating have *n(n-1)* possible dependency links between each other. In principle, this condition is always met in homecare, as at least two participants are always involved: the patient and a health care provider.

(3) **There are uncertainties in the task.** The level of uncertainty does not necessarily affect the need to coordinate. But task uncertainty determines the approach or mode to coordination [18]. Routine task with low levels of uncertainty can be effectively coordinated with impersonal coordination mechanisms like routines, plans, schedules, etc. Non-routine and uncertain tasks require more ad-hoc coordination through communication. Uncertainty can be understood as the number of potential choices in a given situation: the more choices or alternatives (or even possible outcomes), the higher the uncertainty [25,26]. Several types of uncertainty are relevant to this context.

***Task uncertainty*** refers to the difficulty (e.g. complexity, uncertain outcomes) and variability (e.g., same task sequences every day vs. daily changes in tasks) of the work that has to be achieved [18].

***Input uncertainty*** refers to the unpredictability of the task quantity and the task itself, e.g., the unpredictability of the workload or the condition of a new patient [22]. Input uncertainty is high in homecare, as all patients differ regarding diagnoses or comorbidities and care needs, e.g., of those with cognitive impairment, can fluctuate depending on numerous factors [18,22,27].

***Environmental uncertainty*** refers to organization-level physical and social factors that must be considered in decision-making. These factors can be classified according to how simple/complex and static/dynamic they are [26]. The simple/complex rating depends on two sub-factors: how similar the environmental factors are (e.g., all participants or teams belong to one organization); and the number of components involved (e.g., participants, teams, departments, organizations). The static/dynamic scale reflects the extent to which the environment tends to change: no change adds no uncertainty; constant change adds high uncertainty. Based on Duncan [26] description of environmental uncertainty, homecare workers’ environment can be classified as complex-dynamic.

To summarize, the higher the number of dependency links between group members, the more participants are involved, and the higher the uncertainty of the work/environment, the more complex the coordination processes become.

### 3.2 Overview of the coordination literature

#### 3.2.1 The definitions of coordination

Schultz and McDonald [7] found 57 different definitions of coordination in their literature review. They recognized five core elements that the majority of these definitions had in common: (1) involvement of multiple participants; (2) interdependence between the participants; (3) the presence of knowledge about roles and resources between the participants; (4) a foundation in information exchange; and (5) aims to ease the provision of proper health care [7]. Based on these core elements, the authors defined care coordination as

“the deliberate organization of patient care activities between two or more participants (including the patient) involved in a patient’s care to facilitate the appropriate delivery of health care services […]." (p.41)

In other research fields, reviews of coordination (i.e., organizational theory, coordination theory) also cite numerous definitions of the term “coordination” [9,10,17]. For example, Malone and Crowston [17] chose a simple and rather broad definition: “Coordination is managing dependencies between task activities” (p.101). A decade later, working within the field of software development, Espinosa et al. [12] drew from coordination theory sources to arrive at “effective management of dependencies between subtasks, resources (e.g., equipment, tools, etc.) and people” (p.6). Okhuysen and Bechky [9] also found commonalities among the definitions reviewed, namely (1) that people work collectively; (2) that the work is interdependent; and (3) that a goal or task is achieved or completed.

Based on the definitions above, coordination can be seen either as an outcome or a process. A process is a series of actions taken in order to achieve a result (definition of process by Cambridge dictionary 2021). Thus, coordination as a process are the things people need to do to coordinate. The result of these actions is a coordination state or outcome [12]. A good analogy is the difference between what you do to earn money (a process) and how much money you made (an outcome). Coordination as an outcome can be more easily understood when absent. Coordination failures or problems are obvious to anyone, whereas a successfully coordinated outcome may not be as noticeable [17].

#### 3.2.2 Coordination theories and frameworks

In the following section, we briefly describe the terminology of the related frameworks and theories we referenced in our framework development, along with a discussion of their similarities and dissimilarities. We start with those from the extant organizational and coordination research literatures and continue with those that have been adapted or extended specifically for use in healthcare.

Van de Ven et al. [18] described coordination as the process of “integrating or linking different parts of an organization to perform a common set of tasks” (p. 322). They classified this into three types of work activities: impersonal, personal, and group. March and Simon [28], also differentiated between two *modes of achieving coordination* (i.e., as an outcome): through programming and through feedback. Programming is an *“impersonal”* coordination mode involving the proactive application of action plans, rules, standardized information, and systems. Feedback is a *“personal”* coordination mode involving formal and informal, one-to-one and group communication, in response to actions by individuals or groups and focuses more on the actors. Van de Ven et al. described feedback-based coordination as “mutual adjustments based on new information” (p. 323) through either one-to-one or group communication.

Malone [29] introduced *coordination theory* and stated that coordination is “the additional information processing performed when multiple, connected actors pursue goals that a single actor pursuing the same goals would not perform” (p. 5). He classified goal-relevant tasks as either *coordination tasks* or *production tasks*: “Coordination tasks are the information processing tasks that are performed because more than one actor is involved. Production tasks are all the other tasks that are performed in order to achieve the goals” (pp.5-6). In other words, production tasks are needed to complete the task, whereas coordination tasks are needed to work with each other.

Espinosa et al. [12] defined a *coordination mechanism* as “a mechanism that helps teams manage dependencies” (p. 6). They differentiated explicit from implicit coordination mechanisms. Coordination processes are the implementation and use of such mechanisms. Explicit mechanisms are purposely and consciously used by participants to handle task dependencies. Implicit mechanisms are based on “shared knowledge [about the task and the team, which] enables them [participants] to explain and anticipate task states and actions of participants, thus helping them to manage task dependencies” (p. 10). They further differentiate two types of coordination outcomes: coordination as the “state of coordination”, i.e., the extent to which dependencies are effectively managed; and performance or effectiveness, which occurs when key dependencies are successfully managed. These can be regarded as coordination processes and outcomes, respectively. And while Espinosa et al. acknowledge that the verb/gerund *coordinating* and the noun *coordination* are often used interchangeably, they distinguish between the two: “the process of ‘coordinating’ can be defined as the activities undertaken by the participants in managing dependencies” [12]; the state of coordination is the desired outcome of that process.

Faraj and Xiao [22] stated that each *coordination mechanism* contains a specific information processing capability, which needs to be adapted to the information processing requirements of the environment or to the needs from the interdependence of the work units—i.e., not every mechanism is equally suitable for every situation; and *coordinative action* is an unfolding process of linked skills and interconnected activities.

Okhuysen and Bechky [9] adopted the definition of Faraj and Xiao [22] and differentiate between *coordination mechanisms* as organizational arrangements, which enable individuals to perform collectively, and *integrating conditions for coordination*—the “how” behind the mechanism. These authors have not explicitly defined an outcome, their focus within the framework is partly on how coordination occurs and partly on which mechanisms enable it.

And finally, Zackrison et al. [10] distinguish between *coordination mechanisms* (existing structures, objects, processes or interactions to facilitate the coordination of a group or organization), *coordinating* (the organizational process of using coordination mechanisms to achieve a higher level of coordination), and *coordination* as “the extent to which the interactive *in situ* integration of the group(s)' work activities is logical and coherent when it comes to managing interdependencies towards a specific goal” (p. 210). They mention two outcomes: on the one hand, *organizational goals* regarding quality or quantity; on the other, the *reproduction of organizational mechanisms*, knowledge mechanisms, and routines.

Moving on to theories and frameworks in the healthcare setting, Gittell and Weiss [30] do not explicitly define *coordination mechanisms* but distinguish between (1) *coordination mechanisms*, i.e., routines, information systems, meetings or boundary spanners (they integrate work that crosses functional boundaries), (2) *coordination networks*, explained as relationship links—long-term patterns within relationships that serve as channels for resource transfer between actors, and (3) *coordination*, an activity that is essentially about making connections between interdependent actors who need to transfer information and other resources to achieve a goal. As outcomes, they name *quality* and *efficiency of performance*. We argue that with interdependent tasks like homecare, coordination outcomes are antecedents of quality, performance and other final outcomes.

McDonald et al. [31] specify *coordination activities* as actions that are assumed to support coordination. They specify ten such activities: assessing needs and goals, creating care plans, monitoring, adapting, communicating, establishing accountability and responsibility for care tasks, supporting self-management, aligning resources with patient needs, facilitating transitions, and linking the patient to community resources. As an outcome, they name *coordination effects*, which are perceived differently depending on the observer's perspective (system, health care professional, patient), for example, clinical outcomes, utilization-related outcomes or quality of life.

Van Houdt et al. [32] based their framework on 14 key concepts in care coordination. They derived these from a literature review of published theoretical frameworks from various research fields. In their framework they distinguish between *(inter)organizational mechanisms* (i.e., task characteristics, structure, knowledge and information technologies, administrative workflows, cultural factors, required coordination), and *relational coordination* (i.e., roles, quality of relationship, information exchange, goals). They also specify *outcomes* on the *patient level* (including continuity of care or improvements in patients' health status and psychological well-being), the *team level* (membership in a group of specialized health professionals) as well as *organizational level* (care process is performed in an acceptable order and follow each other quickly and smoothly).

And finally, Weaver et al. [13] integrated McDonald et al. [31] coordination activities into Okhuysen and Bechky [9] framework to produce a “Multilevel Framework for Examining Care Coordination.” They mention *proximal outcomes* (i.e., health outcome, care costs, satisfaction, timeliness of care) and *distal outcomes* such as distal health outcomes for individual patients, public health outcomes, lifetime care costs and value. In addition, their framework differentiates between *context* and *setting*, which they classify as either a moderator or input; *coordinating mechanisms* (i.e., approaches, methods, or tools used to align and synchronize care), which they also classify as input; *emergent integrating conditions* (e.g., common understanding, trust) which they classify as mediators; and *coordinating actions* (e.g., communication) which they classify as proximal behavioral processes.

While the various frameworks and theories noted above present disparate views of coordination, all agree that it is a complex phenomenon. Our literature review also noticed a widely-shared conception that coordination includes structures and processes that enable or impede collaborative work, i.e., it ultimately promotes and results in collective performance. We agree with Zackrison et al. [10] and Okhuysen and Bechky [9], who point out that the multifaceted use of the term *coordination* makes it difficult not only to conceptualize but also to operationalize either as a process or as an outcome. Different researchers use different coordination labels and constructs, such as *coordination mechanism*, *coordinating actions* or *activities*, *coordinating*, *coordination*. At the same time, there are some similarities. Conversely, sometimes researchers use the same term to describe or define different concepts. Overall, all the frameworks and theories we reviewed are valuable for research in one way or another. However, none are entirely complete, they each miss some important aspects about coordination, or are too vague to be effectively incorporated into the framework. For coordination to function as a stable concept in healthcare, it is first necessary to understand and define its essential elements and how they are connected.

## 4 Building a theoretical framework for care coordination in home healthcare

In this section we develop our theoretical framework, depicted in Fig. 6, step-by-step. First, we specify a multidimensional concept of coordination as a process, which requires a unified terminology and the various elements associated with coordination processes. We posit that coordination processes are antecedents to coordination outcomes. Next, we differentiate between two types of outcomes: (1) coordination as an outcome, resulting from the coordination process, which is the state of coordination of the group. The group is effectively coordinated if there are no or minimal coordination failures and problems; and (2) patient outcomes, which relate to the accomplishment of the related homecare task's goals. We posit that when the task contains interdependent activities carried out by multiple individuals, effective coordination outcomes are a precondition to patient outcomes. To finish, we discuss the various factors that influence the coordination process.

### 4.1 The multidimensional concept of coordination

Coordination has been described in many different ways in the extant literature. For example, it can refer to actions involving tools that promote, facilitate, or hinder successful coordination outcomes. These can range from physical artifacts (e.g., health records) to abstract or psychological constructs such as group dynamics or respect. It also can denote activities one undertakes (such as communicating) to promote, facilitate or hinder successful coordination; or it can mean the intended effects of such actions (coordination as an outcome). When Malone [29] introduced coordination theory, he explained that it “is in ‘the eye of the beholder' ….The components of coordination are analytic concepts imposed by an observer” (p. 5). However, any scientific discussion of coordination must include a precise shared terminology.

For this work, we adopted the definition of *coordination* used first by Espinosa et al. [12], then by Zackrison et al. [10], as an outcome measure of the extent to which work dependencies are effectively managed towards a specific goal. *Coordination mechanisms* are the “things” in place that promote or facilitate—but, if misused, can also hinder—coordination. The actual actions or activities undertaken to implement or use (consciously or unconsciously) these mechanisms are the actual coordination processes. In a nutshell, coordination mechanisms and processes are what enable participants to manage dependencies [12]. They can also be seen as approaches, methods, or tools available to align and synchronize work [13].

*Explicit coordination mechanisms* are behavioral in nature. They include conscious, purposeful actions people perform to coordinate tasks performed by two or more people (e.g., communicating). In contrast, *implicit coordination mechanisms* are cognitive in nature. These are typically used unconsciously and evolve over time. They involve the knowledge the various participants have about the tasks they are working to fulfill and about each other. Implicit coordination mechanisms enable them to coordinate their efforts with minimal communication [12]. The most effective mix of explicit and implicit coordination mechanisms depends not only on their availability, but also on the structure of the organization (explained further below), the preconditions for coordination, i.e., the types of dependencies, the uncertainties, and the participants preferences [9,12,33]. Table 1 lists our terms and their meanings as we use them in our theoretical framework.

**Table 1:** Definitions of terms denoting coordination elements.

Coordination (outcome)	The extent to which work dependencies are effectively managed towards a specific goal [10,12].
Coordination mechanisms	Mechanisms that help participants to manage dependencies [12,17]. They can be understood as approaches, methods, or tools available to align and synchronize work [13]. We differentiate between explicit and implicit coordination mechanisms.
Explicit coordination mechanisms	Mechanisms consciously used (behaviorally) by participants to help manage task dependencies [12]. Organizational arrangements that enable individuals to perform collectively [9].
Implicit coordination mechanisms	Cognitive mechanisms available to the participants from common knowledge that enables them to explain and anticipate task states and participants' actions and thus to help them manage task dependencies with minimal communication [12].
Coordination process	I.e., coordinating, is the entire process of implementing and applying the necessary coordination mechanisms to achieve positive coordination outcomes [10,12,34].

### 4.2 The dynamic coordination process

As stated in Table 1, the term *coordination process* is understood here as the entire process conducted within a system to achieve a certain degree of coordination. It has to be considered that coordination occurs in different systems. Within the context of healthcare, this includes nursing teams, homecare organizations, or virtually any healthcare setting. Singer et al. [35] differentiate three types of coordination in healthcare: coordination between professionals (or within a care team); coordination across care teams (or facilities); and coordination between care teams and community resources (or support systems). Therefore, coordination can take place intra- or inter-organizationally. Nevertheless, in our view, the coordination process remains the same even if different mechanisms are used and to different degrees. This view is consistent with Gittell and Weiss [30], who observe that the same mechanisms are effective for intra- and inter-organizational coordination.

**Fig. 1:**
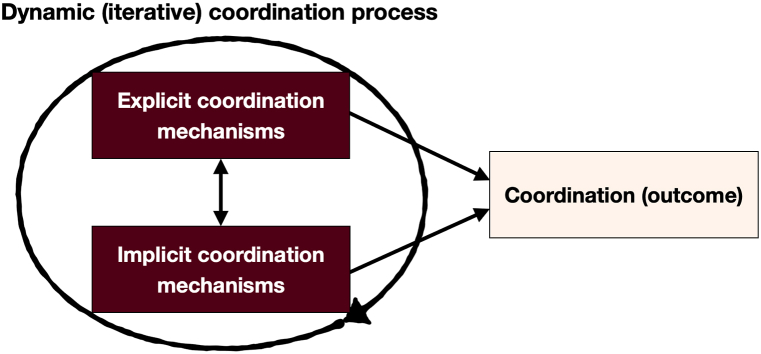
The connections between the coordination process, coordination mechanisms, and coordination.

**Fig. 2:**
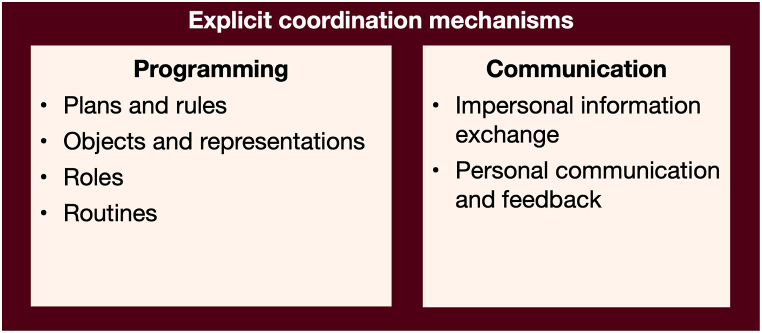
Explicit coordination mechanisms.

**Fig. 3:**
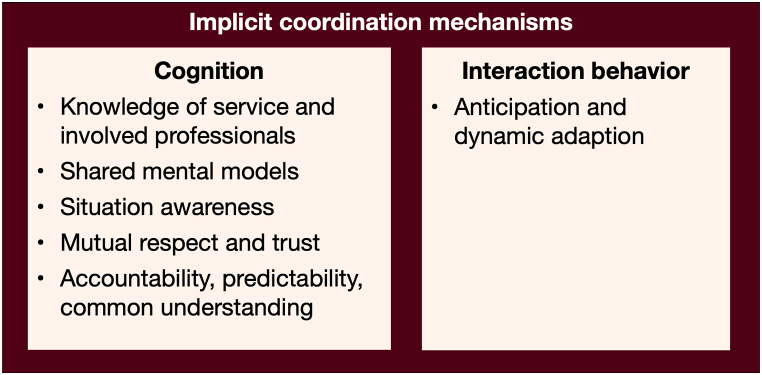
Implicit coordination mechanisms.

**Fig. 4:**
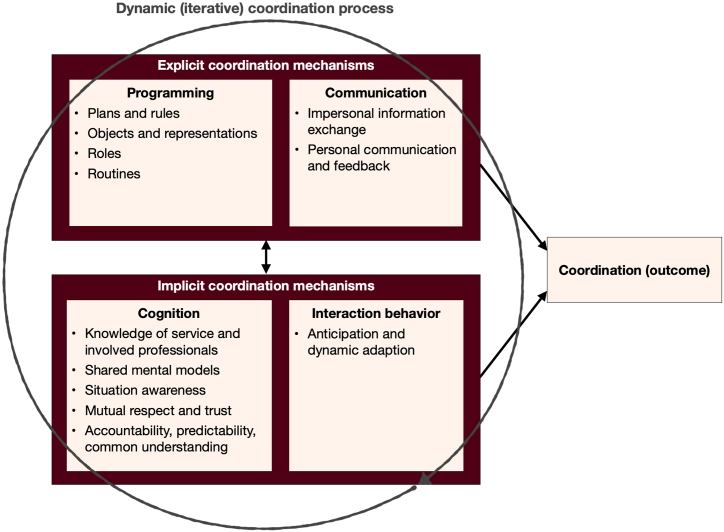
The coordination process with its components.

**Fig. 5:**
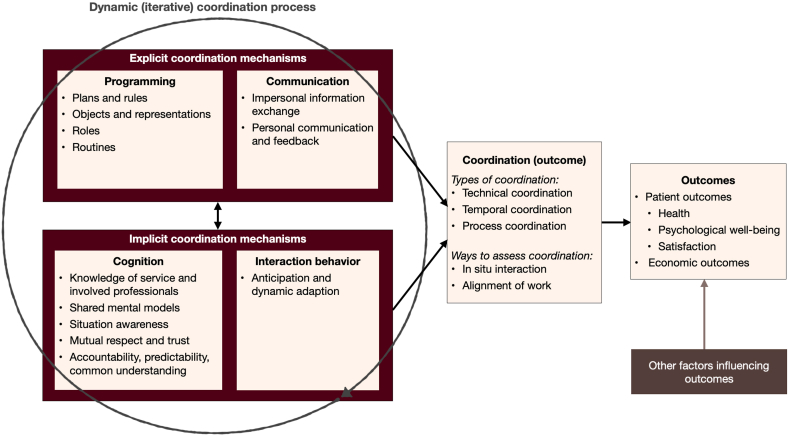
The coordination process, coordination, and outcomes.

**Fig. 6:**
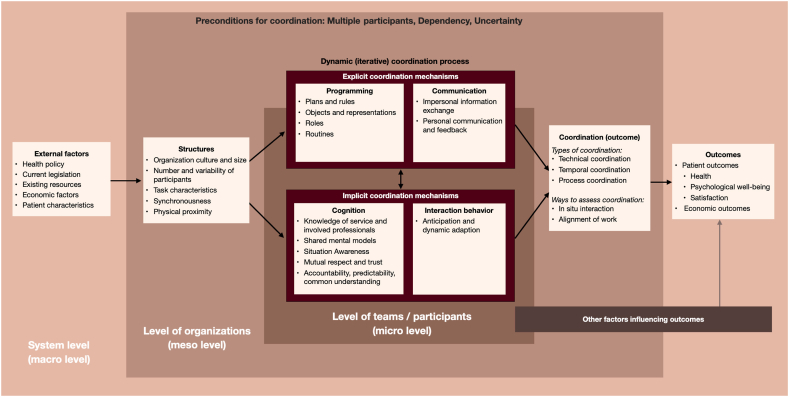
The theoretical homecare coordination framework (Care **COOR**din**A**tion (COORA) framework).

The relationships between the various coordination elements mentioned above are depicted in Fig. 1 (Fig. 1, Fig. 2, Fig. 3, Fig. 4, Fig. 5 pertain to the development of the model shown in Fig. 6).

#### 4.2.1 Explicit coordination mechanisms

Explicit coordination mechanisms can be understood as “mechanisms explicitly used by a team to help manage task dependencies” and are behavioral in nature [12]. More precisely, we see explicit coordination mechanisms as Okhuysen and Bechky [9] see them—as structural arrangements that are purposefully enacted to enable individuals to perform collectively.

These explicit coordination mechanisms can be divided into two categories: *programming* and *communication*. Both have previously been described by March and Simon [28], Van de Ven et al. [18], and Mintzberg [33] and further elaborated in later publications by Espinosa et al. [12], Espinosa and Pickering [36], Rico et al. [37] and Rico et al. [38], among others. Fig. 2 shows an overview of the explicit coordination mechanisms.

##### 4.2.1.1 Programming

Programming is a type of explicit coordination mechanism characterized by blueprints, i.e., detailed sets of information that are impersonally formulated and usually pre-established [12,18]. In essence, programming specifies the division of labor; therefore, it is used to “decouple” or reduce dependencies [33]. These may take time to conceptualize, develop, implement, and learn, but once in place can make coordination of more routine activities quite effective. We use Okhuysen and Bechky [9] categorization, which divides this into the following four groups:

**Plans and rules** include pre-defined plans, schedules, directions for resource allocation (e.g., time, manpower), formalized rules, policies, and procedures [9,12,18,33]. Plans and rules explain the activities required to accomplish a task and provide guidance regarding the work that the various participants must perform [9,13].

For example, a shift or route plan determines when individual people work and when and by whom the patients are to receive care. Plans and rules can also help to match resources to the tasks to be performed. One of their benefits is that they develop commitment between participants [9,13]. To return to the example of shift planning (which is binding), based on guidelines or standards, it determines how much time is available for individual patients and what level of training (i.e., competencies) each nurse on the shift must have. Plans and rules can evolve at the team (micro) or organization (meso) level. In addition to presenting instructions on what needs to be done by whom (e.g., for each new patient admission in homecare), they make it easier for the different participants to relate to each other.

**Objects and representations** include programming items that rely on information technologies (e.g., letters, e-mail, telephone calls/texts, information boards, shared calendars) and patient files (e.g., care plans, electronic patient records, protocols) [9,13,22]. Each object or representation provides a common space for the exchange of information relevant to the participants' task set [9]. For example, if a patient's family doctor orders a change in therapy, that change can be communicated to the responsible nurse through a telephone call or an e-mail.

However, information technologies not only enable the sharing of information but also enhance *situation awareness* (discussed later): by making the various participants' activities visible, they also facilitate the coordination of future work [39]. Representations such as nursing plans or protocols help to operationalize the various tasks and provide a common point of reference that reminds the participants of what they have to do [9,13].

Furthermore, shared protocols are an excellent example of how those involved in a patient's treatment can develop “a common mental model of the patient's condition and the treatment options” [9,22]. Another example is an entry in the patient's record about changes in their health status and any necessary adjustments to their care plan. This informs all subsequent nurses about which aspects of that patient's care they should observe closely or what new interventions they need to carry out.

**Roles** are bundles of defined responsibilities held by individuals. Applying both to staff who work within an individual team, profession or organization and to *boundary spanners* (who work across those groupings), roles facilitate the division of labor. As roles are closely related to expectations associated with social positions, they can also facilitate continuity of behavior [9,40]. Clear definition of both, roles and their associated hierarchies allow the various participants to monitor progress on tasks and to elicit commitment from one another regarding their activities [9,22]. Roles also help to create a common understanding of responsibilities for routine tasks [9]. For example, if individuals understand which tasks are linked to the roles of which care team members, they can replace one another other in the execution of those tasks for which they have the necessary competencies.

For example, homecare nurses are often assigned overall responsibility for a certain number of patient cases. If a patient's situation changes, the responsible nurse must be informed. And as the role of each responsible nurse is clearly defined, other nurses can temporarily substitute for them in case of illness. One specific role to mention here is that of *boundary spanners*. Gittell [27] notes that, by providing information across groups within their organization, they contribute importantly to coordination by clarifying which tasks remain to be done by which teams [9]. One good example of a boundary spanner is a case manager, who must ensure that patients receive adequate care across professional groups or even organizations [27]. In homecare settings, *defined care coordinators* (e.g., nurses or general practitioners) can also take on boundary-spanning roles [41].

**Routines** are “repeated patterns of behavior that are bound by rules and customs and that do not change very much from one iteration to another” [42]. They include handovers, clinical pathways or algorithms, training, regularly scheduled sessions/meetings, or even standardized information and communication systems [9,13,27,36,43]. As the sequence of activities to be performed is well-established, dependent participants can gauge their progress through a routine, as well as knowing when it is complete [9]. Routines also define how and when tasks move from one participant to another (e.g., shift handovers). They may also provide guidance for moments when people work together on a task, e.g., handover reports.

The interpersonal connections included within routines facilitate interactions between participants [9,27,43]. By specifying in advance the tasks to be done and the order in which they are to be performed, they can also help create a common perspective on the pending work [9]. For example, in addition to promoting task agreements, *clinical pathways* can provide insights into the overall care process, the roles of participants, and the level of importance participants place on each of their allocated tasks [27]. Van Houdt et al. [44] found that care process standardization (through care pathways) across the primary and hospital care continuum led to clear definitions of required expertise, roles, and goals. In addition, by diminishing the need for interaction between participants, routines are a relatively inexpensive coordination mechanism [9]. However, the higher the level of uncertainty, the fewer routines are applicable, and the more communication and feedback are necessary [12,18].

##### 4.2.1.2 Communication

Communication can be understood as information exchange or feedback explicitly undertaken by participants when managing dependencies [12,18]. It can be divided into personal and impersonal communication [12,18,37].

**Impersonal communication** refers to a set of impersonal practices and tools that participants use to manage the more stable and foreseeable aspects of work. As well as standardized information and communication, this includes board postings, general announcements, memos to all staff, manuals, written documents [18,37]. As an example, if a nurse documents a pain medication administered to a patient in the health record system, subsequent nurses know what the patient received, even if no personal exchange occurred. Health record system documentation would be classed as impersonal communication.

**Personal communication** involves communication and feedback processes and encompasses the exchange of information between two or more participants to integrate their respective contributions; exchanges can be formal or informal, oral or written [18,37]. Personal communication can be conducted one-to-one or in groups. ***For one-to-one communication****,* the individual participants use vertical (hierarchical) or horizontal (non-hierarchical) interpersonal communication channels (communication and feedback) to coordinate their tasks [18]. The larger the team, the more impersonal and vertical communication is required [18]. ***Group communication*** is used to conduct meetings (whether planned or unplanned) to coordinate tasks. A formal/impersonal tone is common for more routine, usually scheduled communications such as staff or committee meetings; for unplanned communications such as informal, spontaneous conferences between two or more participants about work-related issues, a personal/informal tone is more common [18].

#### 4.2.2 Implicit coordination mechanisms

*Implicit coordination mechanisms* are those that are available to the participants through common or shared knowledge [12]. They help participants cope with task dependencies by being able to explain and anticipate the task states and activities of others, which can help them plan their own activities. These mechanisms are applied consciously or unconsciously and they develop over time through experience, interaction and training together. Thus, implicit and explicit coordination mechanisms influence each other [12].

For example, over time, the homecare nurses learn the physicians' procedural and interaction preferences (an implicit coordination mechanism). It is common knowledge, for example, that physician A is best reached by telephone (explicit coordination mechanism) to discuss patients’ urgent concerns. Fig. 3 provides an overview of implicit coordination mechanisms, distinguishing those that are cognitive from those that are interactive/behavioral.

##### 4.2.2.1 Cognition

**Knowledge of service and involved professionals.** Successful coordination requires an in-depth knowledge of available services and which professionals will be involved in care [16,30,32,[45], [46], [47]]. For example, a homecare nurse needs to be familiar with local services such as meal delivery or financial support options. Before arranging these services for the patient, though, it is also necessary to know which are covered by insurance. There are multiple implicit coordination mechanisms based on cognition, including:

**Shared mental models.** Mental models are “organized knowledge structures that enable individuals to interact with their environment”: they help people to understand and predict events in their environment [48]. *Shared mental models* are based on similarities in knowledge content and structure between individuals, which enable participants to predict their colleagues' information and resource needs [48,49]. The various common mental models can be split into task-based and team-based models [12,48,50].

***Task-based models*** focus on functionality. They can be divided into technology/equipment models (knowledge of technologies and equipment and their limitations, such as electronic health records, prescription systems, and procedures, devices and materials) and task models (knowledge of task sequences, procedures or treatments and relationships between task components, emergency plans, and environmental constraints) [48,49,51].

***Team-based models*** can be divided into team interaction models (knowledge of roles and responsibilities, information sources, interaction patterns, communication channels, role interdependencies, information flow) and team models (knowledge of team members' skills, attitudes, preferences, and tendencies) [48,52]. In order to determine care plans, nurses need appropriate knowledge of their care team members’ experiences, skills, plans, relationships, and preferences. Clinicians involved in the care of a patient may also have different opinions about the roles they and others should play in patient care. Such differences in role perceptions can lead to ineffective navigation back and forth across professions or organizations [46].

Espinosa et al. [12] argued that the importance of the different mental models for the management of interdependencies varies depending on the type of dependency. They also observed that when participants work asynchronously and are geographically dispersed, task-based models seem to play a greater role than team-based ones.

**Situation awareness**. Endsley [39] defines *situation awareness* as “the perception of the elements in the environment within a volume of time and space, the comprehension of their meaning and the projection of their status in the near future,” i.e., “knowing what is going on.” Situation awareness is situation-specific and more dynamic and fleeting than shared mental models, which are more durable knowledge and are not dependent on the situation [53]. For example, knowing a patients preferred walking aid is an example of a shared mental model, whereas knowing what the patient is doing at one particular point in time during mobilization is an example of situational awareness. *Team situation awareness* is up-to-the minute, relevant knowledge required for the participant's responsibilities in a specific situation and is no longer relevant when the situation no longer applies to that participant [39,53]. If a high level of mental model sharing is present, each participant can achieve an equally high level of situational awareness without additional verbal communication [53].

**Mutual respect and trust**. When participants trust and respect each other because they know one another's competencies and expertise, coordination is enhanced [9,54]. Trust also provides assurance that the other participants will fulfill their duties consistently and reliably [9,13,18].

**Accountability.** While shared mental models provide a foundation for common knowledge, a shared sense of accountability clarifies participants' understanding of their and their co-participants’ responsibilities. All participants must be accountable for their contributions [9]. Accountability is vital to ensure that everyone contributes as agreed to the intended “product” [9]. For example, when a colleague hands over a task, the person accepting it must be sure (unless otherwise informed) that all necessary actions have been completed so that subsequent actions can be carried out (e.g., administering the correct amount of insulin prior to meal intake in insulin-dependent patients); if this is not the case, the person handing over the work must take responsibility and inform their successor so that they can plan their activities as necessary.

**Predictability** not only enables participants to anticipate, plan and conduct their own tasks but also gives a picture of subsequent tasks, of any necessary interdependent tasks, and of the entire set of tasks to be accomplished. When predictability is high, participants can be secure in the knowledge that their teammates will successfully perform their work, allowing them to perform tasks that depend on that work as planned [9]. As an example, if the homecare team knows on which day the patient will be discharged from the hospital and what his/her further treatment will be, they can make the necessary preparations (e.g., organize wound dressing materials for home).

**Common understanding** or common ground, enables a shared perspective on the necessary tasks and integration of each individual's work into the whole. A common understanding of the broader context in which coordination occurs, such as the organization's or patient's goals, can keep everyone focused on common patient outcomes. Common understanding enables participants to develop both a common vision and common ground, enabling them to focus their efforts on a shared conception of the work or the processes necessary to complete it [9,55]. Various studies have demonstrated the value of such common objectives [27,30,32].

##### 4.2.2.2 Interaction behavior

*Interaction behaviors*, which Rico et al. call “team situation models,” occur when participants anticipate both the needs of their co-participants and the demands of the task, and dynamically adapt their behavior without planning or communicating directly with one another [23,37]. Rico et al. [37] identify two components within this phenomenon: (1) **anticipation,** which is reflected in participants' expectations and predictions of task demands and expressions of each other's actions and needs without being directly informed of those actions or needs; and (2) **dynamic adaptation**, which is reflected in the actions that participants continuously take to adapt their behavior to each other [37]. This implicit coordination mechanism can be characterized by the following conditions: (1) other participants are provided with task-related information, knowledge, or feedback without prior request; (2) the workload is proactively shared with colleagues or help is offered as necessary and accepted; (3) participants' progress in their activities is monitored; and (4) participants adjust their behavior to match the expected actions or needs of others [37].

Fig. 4 shows the relationship between the various components of the coordination process.

### 4.3 The two different outcomes of the coordination process

#### 4.3.1 Coordination (outcome) – the outcome of the coordination process

As noted above, the coordination as an outcome is understood as the extent to which work dependencies have been effectively managed toward the fulfillment of a specific goal [10,12,17]. Espinosa et al. [56] studied coordination in software development and differentiated three categories of coordination outcomes: *technical*, *temporal*, and *process*. While their study focused on software development, this differentiation is quite general and therefore applicable to other task contexts, including healthcare.

The importance of these outcomes is most obvious when their absence leads to coordination problems or failures. A **technical coordination** outcome is one in which the inherent technical dependencies of the task itself are effectively managed. In homecare, an example would be the successful integration or application of various services or treatments to a patient. Technical coordination failures would include prescribing or administering medication with negative interaction effects or applying a treatment to a patient that leads to severe effects in unrelated medical conditions (e.g., administering in-home tube feeding to a patient who should not receive it due to a diabetic condition). **Temporal coordination** denotes the timely management of sequential dependencies and the transmission of all relevant patient information from one care provider to the next and the timely delivery of the specified health service tasks to the patient when they are needed and in the correct order. An example of a temporal coordination failure would be when a patient receives a prescribed treatment too late for it to be effective, e.g., in a homecare patient with heart failure, increasing diuretic medication too late, leading to a medical emergency because of unchecked pulmonary edema. **Process coordination** focuses on following established procedures and processes, with each participant completing the tasks for which they are responsible in the recommended order and in compliance with established processes and procedures. An example of process coordination failure would be when a minor patient is treated without the parent's consent, or when a patient is released earlier than planned from hospital without involving the homecare organization according to usual procedure, leading to complications and hospital readmission.

However, the distinction between technical, temporal, and process coordination in evaluation or measurement (when failure is not being measured) is rather difficult. This brings us to the framework of Zackrison [34]. In that work, rather than emphasizing different types of coordination, Zackrison proposed assessing coordination based on two more observable phenomena: *in situ interaction* and *alignment of work*. **In situ interaction** can be assessed in terms of accurate and timely information sharing, prompt negotiation of differences, lack of disagreement, and problem-solving capabilities. **Alignment of work** can be assessed based on the degree to which the work is coherent, tasks are not duplicated, all group members perform the tasks they are supposed to do, participants can do their jobs without getting in each other's way, there are no delays in the process and subtasks are closely harmonized.

#### 4.3.2 The outcome of coordination – the patient and economic outcomes

Higher levels of coordination lead to increased performance towards intended results/outcomes [12,13,57]. In healthcare, the target of a care team—whether within an organization or inter-organizational—is to deliver healthcare that meets patient needs effectively and appropriately. The logical consequence of this is that coordination pursues the goal of improving patient outcomes by delivering effective and appropriate care to patient while reducing costs by avoiding empty runs and resource waste [57]. Therefore, with successful coordination in place, both better patient outcomes such as reduced unplanned health care utilization, and better economic outcomes such as reduced cost can be expected [8,13,32,57].

However, it is important to recognize that, while coordination can lead to more effective team performance, it is not always the main driver for delivering effective and appropriate health care. Many factors that impede or improve performance are not related to coordination. Members of a healthcare team may be extremely well-coordinated and still perform poorly. There are two possible reasons for this: First, they may be affected by other determinants of performance that have nothing to do with coordination (e.g., individual or equipment capabilities may not be adequate to perform the necessary tasks, or the patient's condition may be especially complex). Second, some dependencies may affect performance more than others; and while many dependencies may be properly managed, some of the most critical ones may not [12]. For example, even if a healthcare team provides excellent care to a homecare patient and successfully manages all dependencies, if the patient's diagnosis is incorrect, the treatment plan will also be incorrect. In such a case, even a highly coordinated team will perform poorly in terms of treatment outcomes (e.g., complications, worsening of the disease, or even life-threatening conditions).

An additional critical point mentioned by Malone and Crowston [17] is that “often, …good coordination is nearly invisible, and we sometimes notice coordination most clearly when it is lacking” (p.90). This is likely one reason, apart from coordination's vague conceptualization, why many studies of it use outcomes at patient level without measuring – or even considering – measuring coordination as an outcome, i.e., the level of coordination. Fig. 5 depicts the coordination process and its outcomes.

### 4.4 Factors influencing coordination processes

Coordination processes cannot be considered separately from the system within which it works. This is especially true in healthcare. It is essential to consider it in relation to its environment, i.e., characteristics of the meso (organizational) level and factors at the macro or system levels [12,13,32,36,58].

#### 4.4.1 Organizational characteristics at the meso level

Several organizational characteristics affect the coordination process. One general point emphasized by Crowston [59] is that "organizations with similar goals will have to manage the same dependencies, but may choose different coordination mechanisms, thus resulting in different processes" (p. 159).

Factors such as **organizational culture and size** as well as the **number and variability of participants** influence the coordination process [12,16,59]. Organizational culture influences it by determining which coordination mechanisms are in place, for example, how technologies are used or how many tasks are standardized [12,25,33]. Regarding the team, the larger the number of members, the larger (exponentially) the number and complexity of the dependency links between members, resulting in more coordination challenges [12]. The team's composition also plays a role: the participants' experience, types and levels of specialization or expertise, prior experience working with the other members, or the linkages and boundaries between them all influence the coordination process [16,36]. Espinosa and Pickering [36] observed that, based on personal experience and interaction styles, participants prefer certain coordination mechanisms.

Further, the **characteristics of the task(s)** also play a role as they determine what kind of dependency exists [12,59]. The task characteristics of a car manufacturer differ from those of a hospital or homecare organization. Thus, while one organization may operate with a majority of sequential dependencies, another may have to deal with predominantly reciprocal dependencies. The tasks’ complexity, their length and the way they are interrelated also affect the coordination process [16,59,60]. Some tasks are purely digital (e.g., software development) or knowledge-based (e.g., writing a book). Others are mostly physical (e.g., construction, nursing). Furthermore, the task coordination mechanisms and processes themselves may be digital (e.g., email, electronic documents) or physical (e.g., communicating in the operating room).

The coordination process is also influenced by whether the **tasks are synchronous or asynchronous** [9,12]. In an operating room, for example, where tasks are mainly synchronous, members of the surgical team must maintain a high level of situation awareness to work simultaneously; throughout each procedure, all work must be tightly coordinated [39,61]. Performing such synchronous tasks, the participants rely far less on mechanisms such as objects and representations than on their own roles, routines, and mutual trust to know exactly what they have to do and when. For example, situation awareness is critical during emergency surgery or in an airplane cockpit. In contrast, homecare work is largely asynchronous. For each patient, a homecare worker typically works alone, with no team on-site to provide immediate support if needed. The different participants contribute to the patient's care in a time-shifted, i.e., asynchronous manner.

For example, a patient recovering from a leg fracture receives home care services in the morning and evening. In the afternoon the patient goes to the family doctor because the homecare nurse noticed an elevated temperature and an unusual urine smell in the morning. Later in the afternoon, a physiotherapist is scheduled to visit the patient for movement exercises. In the evening, a second nurse will visit. These five participants (the patient, the two nurses, the physician and the physiotherapist) need to be coordinated; but the applied coordination mechanisms and activities they apply differ widely from those of the surgical team. By scheduling the first nurse in the morning, the physiotherapist in the afternoon, and the second nurse in evening, the home care agency has already used coordination mechanisms. When the nurse places a phone call to the family doctor to make an appointment, then helps the patient organize transportation to the doctor's practice, these represent two more coordination mechanisms. The physiotherapist must also be informed (by e-mail) that the patient will not be home at the scheduled time. Assuming that the physiotherapist can move the scheduled therapy session to later in the afternoon, he/she will need to adapt that day's therapy to the patient's condition. The homecare nurse who visits the patient in the evening must then check the patient's condition and, if necessary, organize, adjust or administer any necessary medications.

Another factor that influences the coordination process is **physical proximity**. By facilitating the possibility to see each other face-to-face, physical closeness enables easier exchanges, including informal conversations [9]. For example, when Espinosa et al. [56] examined how working in online environments affected team communication, they found that dispersion has a negative effect on coordination.

#### 4.4.2 External factors at the health care system (macro) level

System-level factors such as **health policy, current legislation, economic factors, and existing resources** also influence care coordination [16,[62], [63], [64], [65]]. Health policy and current legislation influence coordination in numerous ways, e.g., through incentives or financial rewards, by providing definitions, e.g., of various actors' responsibilities, or eligibility criteria for home care patients [[62], [63], [64]]. For example, O'Malley et al. [65] and Williams et al. [64] found that home care agencies saw additional staffing costs to cover administrative and coordination efforts as a barrier to coordination, especially when reimbursement did not cover those costs. Other factors such as workforce adequacy and the sharing of electronic health records are coordination facilitators; workforce shortages and limitations to access to shared electronic health records are impediments [64,65].

**Patient**s have a special role in care coordination. As they are the “consumers” of health care services, their **characteristics** can actually be classified as system-level factors. However, this designation does not give them special privileges: depending on governmental regulations—when eligibility criteria are applied, for example—some can find themselves excluded from services [62]. Similarly, cultural differences (or other dissimilarities) between regions or countries can affect the patients’ social networks or the availability of informal care [47].

Patients can also be placed at the organizational level—depending on the focus of their care-providing organization(s), patient characteristics can vary tremendously. A team (either inter- or intra-organizational) that cares for women after childbirth has a very different patient population one that cares for individuals with cancer or who are at the end of their lives.

However, regardless of which organizational level they occupy, every patient is also the central member of their care team. As such, each also has a duty as an active participant in the coordination process—not simply a passive recipient of services [31,47,64]. Van Houdt et al. [32] and Williams et al. [64] found that, in addition to their individual characteristics—e.g., coping ability, participation in social networks and personality type—patients' expectations are linked not only to coordination needs but also to outcomes. Therefore, as noted briefly above, depending on the perspective chosen, patient characteristics also find a place in the “other factors influencing coordination” category. In our model, we added patients to the system level. Fig. 6 (below) displays the final model.

## 5 Discussion

This paper integrated a broad group of coordination theories and frameworks into a unified, comprehensive theoretical framework that captures care coordination in homecare. The availability of such a framework in the homecare setting will greatly improve the conceptualization and measurement of coordination. This theoretical coordination framework adds value in four main ways: First, based on a wide range of influential coordination literature, the research team has developed the framework iteratively in consultation with healthcare professionals, patients and their relatives, who provided descriptions and explanations to better understand the patient care context. Second, it is comprehensive, considering the complex relationships between the many factors influencing coordination, and applicable across all healthcare settings, not only in the homecare setting. In addition, to the best of our knowledge, it is the first theoretical coordination framework to explicitly address micro-, meso-, and macro-level system factors and their connections within an overarching framework for homecare coordination. Third, our specific explanation of coordination's foundational elements enables a more uniform operationalization of the overarching concept of coordination. And fourth, this framework emphasizes the importance of distinguishing between coordination as a process, coordination as an outcome, and patient outcomes. In measuring as well as in interpreting results this distinction is vital to avoid misleading conclusions.

### 5.1 Limitations

This theoretical framework is conceptual and requires further empirical testing. There are many ways to do this. One important first step would be to evaluate the framework with a qualitative study to better understand the variables that play a critical role and define these accordingly. The results of such a qualitative study could then be used to develop and assess more precise constructs to be tested with quantitative methods. Once we understand how to observe and measure these constructs, the actual framework could then be tested with statistical or ethnographic methods. Once the framework has been tested, we or other researchers could design studies to test individual aspects and components of the framework as a basis for measuring coordination's various elements and their effects. It is important to note that there is an abundance of empirical studies in coordination outside of the healthcare field, so there are many feasible constructs and variables we could employ. Naturally, these would have to be validated in the homecare context. As this framework is comprehensive, measuring all elements might not be feasible; therefore, we recommend identifying the elements on each level (in each box in the model) that are expected to play key roles in each selected setting and are measurable using an appropriate design and validated measurements. Finally, coordination issues vary widely in form and nature across different healthcare settings. Researchers interested in healthcare coordination research need to discern the more generally applicable aspects of our framework and those that may be unique to their focal healthcare context and tasks.

## 6 Conclusions

This framework is thorough and strongly relevant to coordination research in general. Once its application has been tested globally, it will be available to guide researchers to operationalize the concept of coordination in various healthcare contexts. With its potential to standardize our understanding and measurement of coordination, it could also contribute significantly to current practice. Finally, this framework clarifies the critical but formerly muddy distinction between coordination as a process and as an outcome. This distinction will prevent false conclusions or inferences about coordination processes based on product outcome assessments and vice versa.

## Author contribution statement

All authors listed have significantly contributed to the development and the writing of this article.

## Funding statement

Nathalie Möckli was supported by Stiftung Pflegewissenschaft Schweiz (Nursing Science Foundation Switzerland) [ID 3003–2019].

## Data availability statement

No data was used for the research described in the article.

## Declaration of interest's statement

The authors declare that they have no known competing financial interests or personal relationships that could have appeared to influence the work reported in this paper.
